# Here’s the Why and How

**DOI:** 10.1007/s13280-013-0401-y

**Published:** 2013-04-26

**Authors:** Jack Valentin

**Affiliations:** Jack Valentin Radiological Protection, Öregrundsgatan 15, 115 59 Stockholm, Sweden

The final disposal of spent nuclear fuel is a huge undertaking. It raises enormous and highly significant questions, ranging from ethical and philosophical foundations through scientific, technical, financial, political, sociological, and many other issues. Not surprisingly, it attracts profound interest from many quarters.

Thus, we need science and technology, and we also need information. Sweden is fortunate in that long ago, foresighted people realized that disposal would be required, and arranged for the necessary funding. A disposal method has been devised, and an application for a license to use that method is now being scrutinized by the authorities.

Much information is available about this, but different users need different kinds of information. The license applicant, the Swedish Nuclear Fuel and Waste Management Co. (SKB), provides a wealth of easily accessible folders, web pages, and other means of obtaining an at-a-glance understanding. They also provide an overwhelming number of erudite, detailed, and indispensable (but sometimes not all that palatable) technical reports.

Between these extremes, there is a demand for detailed information in a scientific journal with the full rigor of peer review and scientific references, yet reasonably accessible even to seriously interested lay readers. Many scientific disciplines are involved and scientists need information at the edge of their own area of expertise (and honestly, may wish to refresh their own core knowledge). The independence of a scientific journal is particularly important for politicians. The concise format and the careful attention to style of *AMBIO* are helpful for interested laymen. The present Special Issue of *AMBIO* intends to satisfy these needs.

However, it dawned on us at an early stage that the information would be useful in a much wider context than just nuclear waste disposal. In the long run, that could be the most important aspect of this Special Issue, since long-term forecasting of possible developments is required in lots of situations.

As a first stage, SKB invited scientists who had participated in writing technical reports underpinning the license application to a meeting in October 2011. The outcome was that the project would indeed be possible and desirable. An editorial group was convened formally, comprising Ulrik Kautsky (project leader for the initiation, research coordination, and organization of this issue), Tobias Lindborg (project leader for the biosphere site description, “SDM—Site,” and the summing-up phase of the biosphere safety assessment project, “SR-Site”), and myself (independent Guest Editor, former senior regulator, and senior radiological protection adviser).

The Special Issue does not cover every detail of the safety assessment behind the license application. However, we knew in May 2012 that the papers envisaged would form a coherent and adequate description of the assessment. Now that the final product of our labors is emerging, I am grateful, proud, and pleased to have been part of the project. I wish to thank SKB for rendering the project possible, my editor colleagues for many patient explanations and for frequently helping me back on track and schedule, and *AMBIO* for all practical assistance and understanding. In particular, I am grateful to all the authors and the many reviewers without whose diligent efforts this Special Issue would not have been possible.

